# The Mouse Hospital and Its Integration in Ultra-Precision Approaches to Cancer Care

**DOI:** 10.3389/fonc.2018.00340

**Published:** 2018-08-28

**Authors:** John G. Clohessy, Pier Paolo Pandolfi

**Affiliations:** ^1^Preclinical Murine Pharmacogenetics Facility and Mouse Hospital, Beth Israel Deaconess Medical Center, Harvard Medical School, Boston, MA, United States; ^2^Cancer Research Institute, Beth Israel Deaconess Cancer Center, Department of Medicine and Pathology, Beth Israel Deaconess Medical Center, Harvard Medical School, Boston, MA, United States

**Keywords:** mouse models, PDX, precision medicine, cancer, Mouse Hospital, Co-Clinical Trial

## Abstract

Precision medicine holds real promise for the treatment of cancer. Adapting therapeutic strategies so patients receive individualized treatment protocols, will transform how diseases like cancer are managed. Already, molecular profiling technologies have provided unprecedented capacity to characterize tumors, yet the ability to translate this to actionable outcome in the clinic is limited. To enable real time translation of personalized therapeutic approaches to patient care in a co-clinical manner will require the adoption and integration of approaches that facilitate modeling of patient disease. The Mouse Hospital represents an approach that is ideally suited to pre- and co-clinical evaluation of novel therapeutic strategies for clinical care. Patient derived xenograft (PDX) technologies and *in situ* tumor modeling approaches using genetically engineered mouse models (GEMMs) already have a proven capacity to mimic human tumor responses, and their application can deliver invaluable insights into appropriate clinical approaches for individual patients by mirroring human clinical trials using a Co-Clinical Trial project and Mouse Hospital infrastructure. Additionally, the integration of the Mouse Hospital with other emerging technologies for the application of precision medicines, including organoid technologies, provides a platform that enables medical centers to truly reap the benefits that precision medicine has to offer.

Precision medicine has long been lauded to deliver the cure and eradication of diseases such as cancer, tailoring treatments to the specific genetics and needs of the patient. Indeed, the technological advances that we have seen over the 20–30 years have allowed us to profile and characterize patients and tumors to an unprecedented level, enabling a detailed mapping of genomic alterations and characterization of mutations observed in disease. Yet, the translation of these findings and approaches to the care and treatment of individual patients falls far behind the trailblazing advances in the technology that individualizes tumors. Much of this lag in translation to the clinic, lies in the historical approaches and methods in place for the testing and clinical evaluation of agents to be brought to the clinic, with a lack of infrastructure to facilitate translational studies in academic medical centers.

Our lab has pioneered the development and implementation of The Mouse Hospital and Co-Clinical Trial Project (1–4). This concept offers a mechanism by which tailoring of treatments and design of patient specific therapies can be rapidly evaluated. The Mouse Hospital encapsulates an infrastructure by which mouse trials can be carried out in a manner that mimics human trials and treatments. In this setting, resources including imaging, treatment and pathology mirror human resources, and are integrated with standardized operating procedures and ongoing training of technical staff to ensure best practice and provide a recognized “standard of care” in mice that mimics human treatments. This in turn enables Co-Clinical Trials to be carried out in mice, whereby concurrent human/mouse trials mimic and inform one another. However, such an approach requires a number of important considerations from a practical perspective (3), and its integration within the context of a clinical trial and translational framework to benefit patients requires careful consideration. Indeed, the variety of models and their application offer a number of different and unique approaches that can be adopted and tailored to patient needs, and should be considered in the context of other precision medicine based technologies that offer the potential to identify unique therapeutic protocols for patient treatments. Here we outline key elements of the Mouse Hospital and the Co-Clinical Trial approach that can meet the needs of precision medicine, and discuss the challenges facing these approaches that need to be met to facilitate routine incorporation and utilization to deliver superior cancer patient care.

## Modeling patients in mice

For the purposes of modeling human cancer in mice, there are currently two predominant approaches utilized. One represents the growth and expansion of tumor tissue in immunocompromised mice in a patient derived xenograft (PDX) or avatar setting, while the other represents genetically engineered mouse models (GEMMs), whereby the mouse genome is engineered to harbor key genetic alterations to drive tumor development *in situ* for the purposes of following tumor initiation and progression (5, 6). PDX tumor models have the advantage of studying human tumors themselves, enabling the expansion and evaluation of multiple single agent and combination therapies, however their immune compromised state fails to fully recapitulate the tumor microenvironment within which tumors exist. Although GEMMs may not always fully recapitulate the full heterogeneity and complex genetics of human patients, they do have the advantage of their *in situ* localization, and enable study of evolution and immune related function on tumor growth, progression and response to therapy. This is of particular importance in the context of immune-therapies, which represent a rapidly growing area for therapeutic intervention in many cancer types, and where novel immune targeting therapies require pre-clinical evaluation (7). Indeed, although both PDX and GEMM models have provided important tools for the study of human cancer, there are key challenges that still need to be met in order to provide a more robust and useful platform for integration with clinical studies.

While PDX models offer the opportunity to uniquely match individual patients with mouse avatars for evaluation of drug response to their unique tumor, not all patient tumors grow and progress in a xenograft setting (8–10). In addition, orthotopic vs. subcutaneous tumor implantation for development and propagation remains an issue of discussion (8, 10). Much of the work to date has focused on subcutaneous propagation of PDX tumors, providing easy access to follow tumor growth and monitor response to treatment, and while there is evidence that orthotopic propagation of PDX tumors may facilitate some tumor types, this frequently requires much greater technical expertise, and does not always lend easily to enrollment of tumors at similar stages, and the longitudinal monitoring of individual tumor types. More recently, evidence highlighting the limitations of PDX models to faithfully model human tumors has demonstrated that propagation of human tumors in mice can result in a distinct evolution of these tumors (11). Indeed, while this study noted that the degree of genetic instability between human tumors and PDX models shares similarities, the distinct copy number alterations (CNAs) that occur in the evolution of human vs. PDX tumors highlights how the murine environment promotes clonal selection distinct from that occurring in patients (11). This may have important implications for the reliability of PDX tumors as avatars for human disease and their use in co-clinical studies, particularly as arm-level CNAs can be associated with drug response, and clonal selection resulting from PDX propagation can impact CNAs present, and in turn influence therapeutic outcomes (11).

GEMM models have their own particular challenges and are somewhat limited by the number of genetic alterations and time required for development of tumors *in vivo*. This frequently prevents GEMM models from acting as individual patient avatars, but they approximate patients based on key genetic drivers or modifiers for a particular cancer type. However, the emerging role of immune cell types in cancer has highlighted the need for models to study and understand the relationship in cancer (7). Particularly in the context of therapy where agents targeting immune cells are emerging as key elements for cancer therapy, and increasing relevance of cancer vaccines in maintaining remission and preventing recurrent disease is gaining momentum (12–14). Indeed, GEMM models are also now highlighting how the genetics of the tumor can influence the immune landscape of tumors, and in doing so influence the tumor biology (15). However, adaption and refinement of GEMMs is required to better facilitate pre- and co-clinical trials in the context of the mouse hospital so as to provide a more off-the-shelf approach for their utilization and application to real-time patient trial integration. Abilities to more easily modify genomes utilizing CRISPR genome editing approaches are facilitating this transition, and new opportunities for the development of models and their application are emerging (16–20).

Thus, PDX and GEMMs models complement each other in what they have to offer the cancer patient. A combination of efforts that take into account the patients own tumor, with its heterogeneity and complex genetics, in addition to a more simplified and focused model that looks to the main genetic drivers to account for generalizations amongst tumor types, and that take advantage of both immune-compromised and -competent settings.

## Integrating mouse studies with clinical care

**Figure 1:**
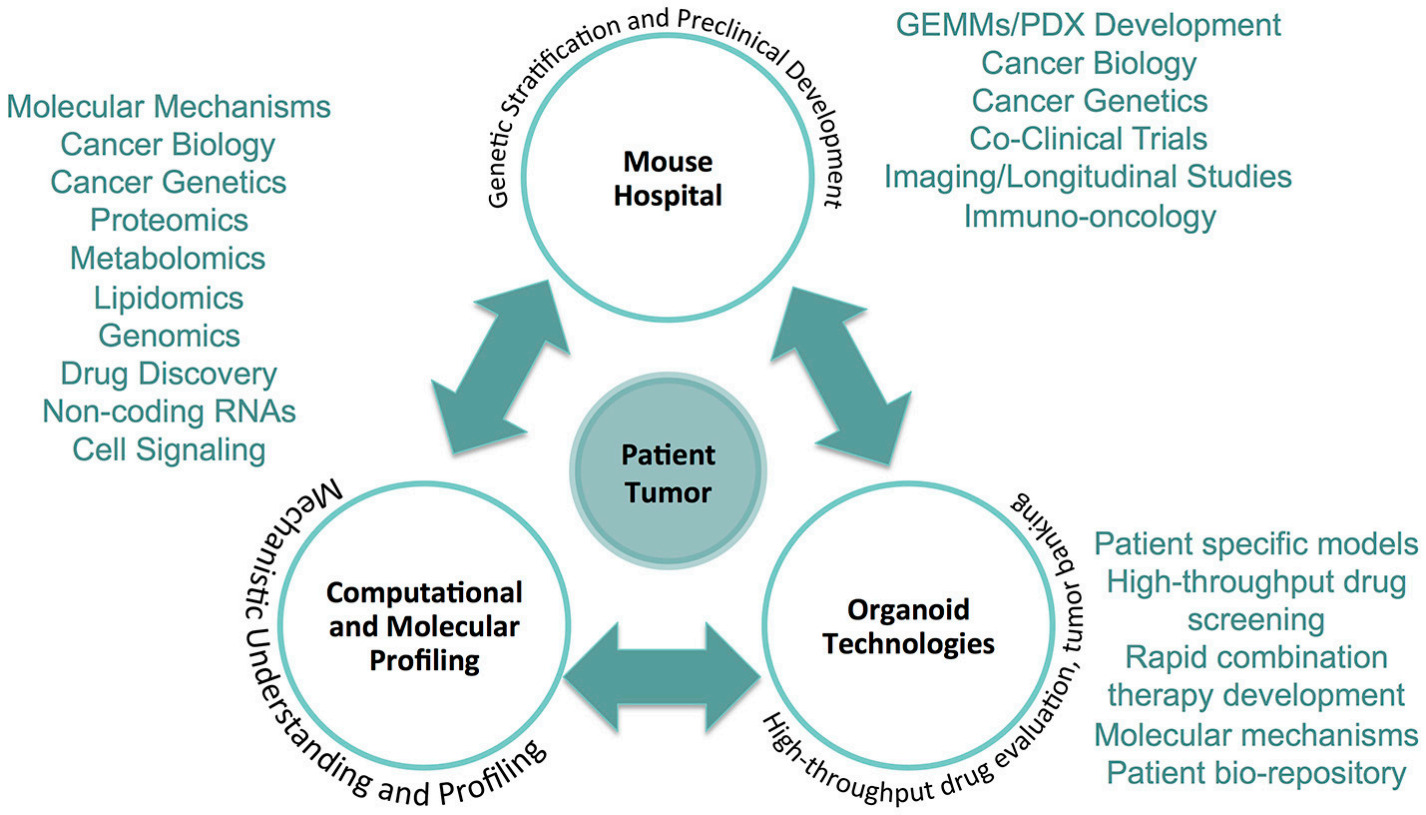
The precision medicine framework integrating The Mouse Hospital. The Mouse Hospital ideally is integrated in an ultra-precision medicine framework whereby data collected from computational and molecular profiling of patient tumors, in addition to screening and characterization of primary cancer organoid cultures, enable prioritization of novel therapeutic strategies for *in vivo* pre- and co-clinical evaluation. The integration of each of these approaches provides a comprehensive platform that can deliver actionable therapeutic strategies for individual patients that goes above and beyond what is currently available based on targeted genomic sequencing.

While efforts aimed at improving models to provide enhancement in their application to uncovering novel therapeutic approaches for cancer is ongoing, how these models inform patient care, and how they are integrated into the precision medicine framework is also of relevance (Figure 1). This requires integration of mouse modeling approaches with existing technologies that have been built to support patient care in the context of precision medicine. Of particular relevance are areas of cellular profiling related to DNA and RNA sequencing, proteomic, and metabolic analyses, as well as culture of primary tissue explants from cancer patients.

In the context of molecular profiling, advances in computational approaches to defining individual patient drug resistance or sensitivity have greatly improved. Large publicly available datasets that include The Cancer Genome Atlas (TCGA), Cancer Target Discovery and Development (CTD^2^) database (21, 22), and the Cancer Cell Line Encyclopedia (CCLE) (23) have already demonstrated both the ability of molecular profiling to identify and hone in on key networks and pathways that provide insights on heterogeneity of tumors and facilitate identification of tumor sub-types, as well as highlighting how a central repository for datasets can facilitate analysis. Enabling data to be stored and input in a central repository can greatly facilitate co-clinical efforts involving multi-center co-clinical trials. Such a resource can extend beyond data sharing and analysis to also include shared protocols, relevant metadata, tools, and greatly facilitate research through web accessibility. Indeed, relevant DNA or transcriptome profiling for patients and models can enable comparison with big-data repositories to identify similarities with tumors already demonstrated to be sensitive or resistant to known chemotherapies or targeted agents (24). Such approaches facilitate the identification of focused therapeutic options for patients, and enable identification of targeted agents appropriate for defined genetic cancer types. In adapting such approaches, the evaluation and divergence of PDX models in particular from the original primary tumor should be followed, comparing CNA and transcriptomic profiles to account for clonal selection through PDX propagation. Indeed, it is possible that computational approaches can “correct” for responses in such situations, providing a statistical framework to facilitate translation of PDX response and outcome in co-clinical studies, to account for such divergence.

In general, while the long latency to generate and propagate PDX models for co-clinical studies provides a challenge for real-time application in this setting, organoid technologies are emerging as an efficient method by which to rapidly grow and expand primary tumors in culture. Current efforts to characterize these cultures as tumor models has highlighted their potential in study and evaluation of their representative patients (25–28). The ability to grow these primary tumors *in vitro*, enables a more high-throughput screening of individual patient tumors for sensitivity to drugs already approved for clinical use or under clinical evaluation. In addition, human organoid cultures can be utilized for the development of PDX tumors *in vivo*. and organoids derived from mouse primary tissues and cell types can provide useful models for human cancer (29–31). Thus, organoid approaches can facilitate with evaluation of tumor sensitivity to therapeutic agents, and enable the testing of multiple combinations of therapeutic agents to identify potential therapeutic strategies for the treatment of individual cancers.

Combining these approaches as first line co-clinical efforts aids in optimally integrating mouse model approaches for precision medicine. Indeed, such *in silico* and *in vitro* analysis enables a well-defined prioritization of therapeutic strategies that can be evaluated and validated *in vivo*. This streamlines the use and application of mouse modeling approaches for translation of novel therapeutic strategies to the clinic, and enhances the effectiveness of *in vivo* translation. Such a pipeline represents an attractive model for the execution of precision medicine for cancer patients, going beyond a simple genetic or transcriptome profiling approach to stratify patients for therapy, to providing an ultra-precision platform that tailors treatments to provide the most optimal therapeutic strategy.

How co-clinical and clinical efforts are integrated and inform one another is also of relevance. The use of patient material for mouse related studies in the context of pre- and co-clinical requires approvals from both Institutional Review Boards (IRB) and Institutional Animal Care and Use Committees (IACUC). Additionally, challenges surrounding patient privacy and how data generated and analyzed are stored needs to be carefully considered, and protocols implemented need to adhere to appropriate HIPPA guidelines if such studies are to directly impact patient care. This requires that institutional infrastructures be in place to ensure that data are properly protected and patient identification only accessible by appropriate clinical staff. Similarly, while more general studies carried out using GEMM models or de-identified PDX models within a co-clinical setting to evaluate response to novel therapeutic agents or combinations thereof, it is crucial that therapeutic response in these models be carefully correlated with relevant response in human patients as outlined below. This frequently requires that individual models are carefully optimized to ensure standardized application of the model to anticipate therapeutic outcome.

## Ultra-precision mouse models for cancer care

Although efforts to utilize mouse models in such an integrated ultra-precision platform is an attractive approach to maximize efficacy of data generated from *in vivo* studies, and provide effective clinical approaches to treat cancer patients, it is essential that strict procedures and protocols are in place to ensure reproducibility and reliability across the platform (3, 32). Currently there are no clear guidelines for how mouse models should be integrated into translational studies that directly impact patient therapy, and several studies highlighting issues concerning reproducibility across academic research demonstrate the need for systems that ensure the reliability of such data. Thus, it is essential that standard operating procedures (SOPs) are generated and in place to provide appropriate quality systems founded on good laboratory practices (GLP) that include detailed protocols, reporting and archiving to ensure all relevant data are recorded for reference and repeatability. It is also important that quality assurance units be included as part of the systems in place to ensure conformation with GLP. Such a GLP approach ensures uniformity and consistency in the performance of relevant studies, and facilitates evaluation of systems in place by regulatory authorities. Indeed, the Organization for Economic Co-operation and Development (OECD) already provides guidelines for testing and evaluation of chemicals that can be readily adapted for co-clinical use (33, 34). In addition, it is essential that appropriate education and training are provided to those carrying out such studies, and that records and data are appropriately maintained and archived. Ultimately, pre- and co-clinical studies involving mice will be carried out in a Clinical Laboratory Improvement Amendments (CLIA) approved environment to facilitate the approval and translation of studies from the bench to the bedside.

It is also important that such approaches be considered and evaluated by internal review boards (IRB), who oversee and approve ongoing clinical trial protocols within the academic medical setting. The ability to inform patient care in real-time, through precision medicine approaches offers unique opportunities for cancer patients, and translating novel therapeutic strategies to the clinical for individual patients based on a cohort of pre- and co-clinical studies requires careful evaluation to ensure patients are protected and offered treatments that truly represent best-option strategies for their specific cancer. In translating these results, the ability to match or predict how response in mouse models equates to a response in human patients is of great importance. The use and application of RECIST (Response Evaluation Criteria In Solid Tumors) and irRECIST (immune-related RECIST) criteria in patients has become an essential set of rules that define patient response to treatment. Equating responses in GEMM and PDX models to appropriate RECIST responses in patients can require optimization and may be developed through iterative processes, but can dramatically improve the clinical relevance of co-clinical studies. This requires that pilot studies be carried out to properly establish an appropriate treatment regime corresponding to patient treatments, which almost invariably includes upfront standard of care therapies. It is therefore important not to jumpstart the process by solely evaluating experimental therapies, but always evaluating and correlating mouse model response to standard of care as appropriate for the relevant clinical trial. This in turn can set clear criteria for evaluating response and subsequently be utilized to support the use of such models in clinical trial protocols, again however, clear criteria and GLP approaches are necessary to ensure reproducibility and reliability as outlined above.

As part of such an approach, it is imperative that drugs and therapies used in the pre- and co-clinical setting mimic as closely as possible those that will ultimately be administered to patients. However, it may not always be the case that such agents can be easily assessed, particularly in the case of GEMMs. Many human specific therapies, including biologics or small molecule inhibitors, demonstrate limited cross-reactivity or specificity for mouse targets, and thus lack of efficacy in such models requires that mouse specific reagents be generated (35). However, the enrollment of mouse models for the purposes for testing of novel therapeutic approaches can greatly facilitate evaluation of both targeted agents, and evaluation of combinatorial therapies. This can greatly aid rapid stratification and testing of multiple therapeutic options, in turn tailoring therapies for patients. Similarly, it is important to consider dosing strategies for corresponding mouse and human trials, and integration of mouse models can provide insights on differential dosage as well as metronomic therapy approaches for clinical application. Indeed, a number of studies have demonstrated the usefulness of mouse models in optimizing dosing strategies for patients to deliver more effective responses to standard cancer therapies (36, 37).

## Future directions

As outlined above, despite extensive advances in technologies that support cancer patients and the ability to characterize their unique cancer, there is a critical need to go beyond utilization of this resource as simply a diagnostic or prognostic tool. While currently such data provides an actionable therapeutic option in limited cases, often reserved for specific targetable mutations, all too frequently much of the information gleaned provides little therapeutic value (38). Thus, the integration of such data with computational and molecular databases, combined with *in vitro* screening and characterization of primary disease utilizing organoid technologies, can be readily translated to the clinic through *in vivo* validation using mouse models (2, 4, 39). It is also of paramount importance to include global genomic and transcriptomic analysis toward more accurate predictions, as well as for the identification of novel mechanisms of resistance as recent studies indicate (40). Development of such a platform to integrate patient and mouse hospitals through co-clinical studies provides a clear pipeline for delivery of ultra-precision solutions for individual cancer patients (41). Such an approach requires careful organization and set-up to ensure such models provide accurate insights for development of patient care strategies and represent a key component of precision medicine centers of the future.

## Author contributions

JGC and PPP conceived, researched, and co-wrote the manuscript.

### Conflict of interest statement

The authors declare that the research was conducted in the absence of any commercial or financial relationships that could be construed as a potential conflict of interest.
